# Informal care and the impact on depression and anxiety among Swedish adults: a population-based cohort study

**DOI:** 10.1186/s12889-021-11246-1

**Published:** 2021-06-29

**Authors:** Marlene Stratmann, Yvonne Forsell, Jette Möller, Yajun Liang

**Affiliations:** Department of Global Public Health, Karolinska Institutet, Tomtebodavägen 18A, 171 77 Stockholm, Sweden

**Keywords:** Informal caregiving, Cohort, Depression, Anxiety

## Abstract

**Background:**

As the population is ageing, the need for informal caregivers increases, and thus we need to know more about the effects on caregivers. This study aims to determine both cross-sectional and longitudinal associations between perceived limitation of informal caregiving and mental health of caregivers.

**Methods:**

This population-based cohort study was based on the Swedish Psykisk hälsa, Arbete och RelaTioner (PART) study, and 9346 individuals aged 18–65 were included. Data were collected through questionnaires, interviews and Swedish registers. Informal care was defined as care given to a family member. Self-reported and diagnosed depression and anxiety were included as outcomes. Covariates included sex, age, social support and socio-economic position. Ordinal logistic regression and Cox regression were performed to determine the associations between caregiving and anxiety or depression.

**Results:**

Self-reported depression and anxiety was only increased among those experiencing limitations (adjusted odds ratios [aOR] 2.00, 95% confidence intervals [CI] 1.63–2.47 for depression; aOR 2.07, 95% CI 1.57–2.74 for anxiety) compared to those not giving care, respectively. The adjusted hazard ratio (aHR) were increased for diagnosed depression (aHR 1.97, 95% CI 1.27–3.05) and for diagnosed anxiety (aHR 1.86, 95% CI 1.06–3.25) among those giving care and experiencing limitations, compared to those not giving care. No significant associations were found in caregivers without limitations.

**Conclusion:**

Caregivers experiencing limitations showed a significant association with short- and long-term anxiety and depression. This study implies the importance of exploring the degree to which informal caregiving can be provided without adding burden to caregivers.

**Supplementary Information:**

The online version contains supplementary material available at 10.1186/s12889-021-11246-1.

## Introduction

Informal caregiving is defined as the care given to a family member (family caregiving), friend or neighbour including emotional and practical help, and is typically unpaid [1]. Furthermore, informal care is usually home-based [1]. In contrast, formal care is a paid service provided by trained healthcare professionals that can be carried out as home-based care, community-based care or residential care [2]. Both informal and formal care include assistance in personal and clinical care and homemaking [2].

According to the Organization for Economic Cooperation and Development (OECD), one in three individuals aged 50 years and above are giving informal care to some degree [3], which complies with the estimates of the European Social Survey Round 7 [4]. In Sweden, the estimates of persons over the age of 18 years that provide informal care differ from 20% up to 29% [1, 4]. As of now, on average 60% of all care in EU countries has been provided by informal caregivers in 2012, with variations across countries [5]. Based on a changing demography with less young people and more elders [3, 6–9], the need for informal caregiving will increase in the future [10]. Furthermore, European governments are retreating from the responsibility of home care and with a smaller proportion of young people being in the work force compared to a bigger proportion of retired people, the labour market might be unable to handle the challenge of an increased need for informal caregivers [9, 10]. Estimations suggest a future deficit of 20,000 informal caregivers in the Netherlands and 400,000 in Germany by 2060 [8].

While the most common reason of informal caregiving is long-lasting illness or disability in older family members, such as dementia, it can also lead to illness in the caregiver her- or himself [10, 11]. Even though studies have reported on positive outcomes of informal caregiving such as an increased positive appraise and occasionally a better well-being [7, 12], informal caregiving has mainly been associated with worse physical and mental health, such as depression, anxiety and burn-out [2, 8, 13–18]. Most studies regarding informal caregiving and mental health focus on the state of mental health in general or perceived stress in the informal caregiver [3, 17, 19, 20]. It has been stated that in OECD countries informal caregivers suffer 20% more from a mental illness than non-caregivers [3].

Generally, informal care is mostly provided by women, although the proportion of men has been increasing in the last years [2, 21, 22]. Especially as the carers grow older, the proportion of men providing informal care increases [3]. In Sweden, the proportion of women and men providing informal care is equal [1, 23]. Yet, the tasks carried out by men and women are different [1]. While women provide help with personal care and serve as interlocutor, men usually provide financial support and practical help [1]. Additionally, previous research has shown that women experience mental ill health, such as depression and anxiety, up to two times more often than men in general [6, 19–21, 24, 25].

So far, it is unclear to what extent informal caregiving impacts anxiety and depression. The association between informal caregiving and anxiety has often been implied in reports and reviews, however, there are very few population-based studies estimating the association between informal caregiving and risk of anxiety and depression [6, 8, 17]. Two reports suggest an increased risk of mental ill-health if participants provide informal care, and one study estimated the same association but could not conclude a statistically significant association [6, 8, 17]. In addition to the duty of giving care itself, perceived limitations due to caregiving, referring to the negative influence on the personal life of caregivers, are hypothesized to be the crucial underlying reasons for mental illness of caregivers. So far, the evidence is lacking on the effect of perceived limitation of caregiving on the mental health outcomes. Thus, the aim of this study was to determine both cross-sectional and longitudinal associations between perceived limitation of informal caregiving and mental health of caregivers.

## Methods

### Study design

Data were used from the Swedish PART (In Swedish short for: Psykisk hälsa, Arbete och RelaTioner) study, which is an ongoing longitudinal cohort study. Based on the PART study, a cross-sectional study design was used to estimate the self-reported outcomes from wave 1 (baseline) and a longitudinal cohort study design was used to estimate clinically diagnosed outcomes and longitudinal self-reported outcomes from the follow-up questionnaire (wave 3). This study included data from wave 1 (1998–2000), wave 3 (2010) and Swedish registers (1998–2014). Those who answered the questionnaire in wave 1 were followed up in wave 3 and afterwards linked to Swedish inpatient and outpatient registers and the cause of death register.

### Study participants

At baseline, a total of 19,457 persons were invited to participate and 10,345 responded (53%). The participants were more likely to be female, older, with higher education and income, born in the Nordic countries and without previous diagnosis of psychiatric conditions [26]. The study population (wave 1) included 10,345 participants aged 18–65 years old living in Stockholm, Sweden. After excluding 18 participants with missing information on caregiving, 7 with missing date of entry and 974 that reported previous depression or anxiety in the baseline questionnaire, the final study population included 9346 participants at baseline for the cross-sectional analyses. Of those, 5108 answered the follow-up questionnaire 10 years later and were included for the longitudinal analyses.

### Informal caregiving

The exposure was defined as informal caregiving to a family member and assessed according to a positive response to the question “Are you currently responsible for the care of a long-time sick or disabled family member?” In order to assess the perceived limitations that informal caregiving can have on the life of the caregiver, three follow up questions were asked regarding conflicts with work, leisure time, and family or friends due to informal caregiving. The exact questions asked were: Are your opportunities for work or leisure activities limited by this care responsibility? Are your opportunities for spending time with friends and family limited by this care responsibility? Does your responsibility for care lead to conflicts with family or friends? The response alternatives included: no, yes to some degree, and yes to a high degree. Based on this, three exposure categories were defined: no caregiving, caregiving with no limitations and caregiving with any degree or dimension of limitations in the caregiver’s life.

### Depression and anxiety

This study focuses on depression and anxiety as outcomes. Using the major depression inventory (MDI), self-reported depressive symptoms were classified into four categories: no depressive symptoms (< 20), mild (20–24), moderate (25–29) and severe (> 30) depressive symptoms. Anxious distress was assessed by using the DSM-5 criteria consisting of five questions from three different scales [27]. These questions were related to feeling keyed up or tense, feeling unusually restless, difficulty in concentrating because of worry or fear of losing self-control, fear that something awful might happen and fear of losing self-control. Self-reported anxious distress was categorised into mild and moderate/severe as severe anxious distress requires a clinical observation of motor agitation [27].

Diagnosed depression was defined as having a diagnosis of either major depressive disorder with a single episode or recurrent depressive disorder (ICD-10 codes: F32–33) in the Swedish in- and outpatient register. Clinically diagnosed anxiety was defined as either having phobic anxiety disorders or other anxiety disorders (ICD-10 codes: F40–41) in the same registers as mentioned previously.

### Covariates

Covariates included age, sex, social support and socio-economic position. Social support was categorised into yes and no and assessed whether the participant had one specific person they felt supported by. According to statistics Sweden, socio-economic position was grouped into categories containing unskilled or semi-killed, skilled, assistant non-manual, student, age or early retirement, self-employed, employed, unemployed or on sick leave or on leave, and others that contain part-time work and duties [28]. Information about the covariates were based on information from the first wave of PART.

### Statistical analysis

Descriptive analysis was reported as mean (standard deviation) for continuous variables and as number (percentage) for categorical variables. The follow-up time started from the date of receiving the questionnaire and ended at the date of outcome, death or 31. December 2014, whichever came first. To determine the associations of informal caregiving with depression and anxiety, two different methods were used. For the self-reported depression and anxiety at baseline and 10-year follow-up, ordinal logistic regression models were used to determine odds ratio (OR) and 95% confidence interval (CI). For the diagnosed depression and anxiety, Cox proportional hazard regression models were used for the estimation of hazard ratio (HR) and 95% CI. Two models were reported from both the ordinal logistic regression and the Cox proportional hazard regression analyses: model 1 was crude, and model 2 was adjusted for sex, age, socio-economic position and social support. In addition, baseline self-reported depression and anxiety were further adjusted in the ordinal logistic regression models for the 10-year follow-up analysis. A sensitivity analysis using cox proportional hazard regression was performed, restricting analyses to those participants that participated in both the baseline and 10-year follow-up to check if the loss to follow-up had an effect on the associations. All analyses were carried out in the statistical programme Stata, version 16.

## Results

As shown in Table 1, the proportion of women was highest in those giving informal care and experiencing limitations. Informal caregivers in general were older compared to participants that did not provide informal care. Social support was more common among participants that did not give care and those that gave care but did not experience limitations. The study participants that gave informal care were more often retired or unemployed.

**Table 1:** Baseline characteristics of the study population by informal caregiving (*n* = 9346)

	No caregiving (***n*** = 8562)	Caregiving without limitations (***n*** = 312)	Caregiving with limitations (***n*** = 472)
Age, years, mean (SD)	41 (12)	48 (11)	46 (11)
Sex, *n* (%)			
Man	3972 (46.4)	134 (42.9)	152 (32.2)
Woman	4590 (53.6)	178 (57.1)	320 (67.8)
Social support, *n* (%)			
Yes	8072 (94.3)	296 (94.9)	438 (92.8)
No	490 (5.7)	16 (5.1)	34 (7.2)
Socioeconomic position, *n* (%)			
Unskilled/semi-skilled	164 (1.9)	4 (1.3)	11 (2.3)
Skilled	54 (0.6)	2 (0.6)	9 (1.9)
Assistant non-manual	142 (1.7)	5 (1.6)	15 (3.2)
Student	639 (7.5)	8 (2.6)	25 (5.3)
Retired	408 (4.8)	22 (7.1)	34 (7.2)
Self-employed	607 (7.1)	23 (7.4)	35 (7.4)
Employed	5402 (63.1)	200 (64.1)	264 (55.9)
Unemployed/sick leave	726 (8.5)	28 (9.0)	47 (10.0)
Others	420 (4.9)	20 (6.4)	32 (6.8)

The prevalence of self-reported depressive symptoms was 8.50% for mild depressive symptoms, 4.23% for moderate depressive symptoms and 7.41% for severe depressive symptoms at baseline and 3.71% for mild depressive symptoms, 1.84% for moderate depressive symptoms and 3.04% for severe depress depressive symptoms at 10-year follow-up. The 15-year cumulative incidence for clinically diagnosed depression was 2.63%.

For self-reported anxious distress, the prevalence was 5.2% for mild anxious distress and 3.3% for moderate to severe anxious distress at baseline. At 10-year follow-up the prevalence was 2.02% for mild anxious distress and 1.22% for moderate to severe anxious distress. The 15-year cumulative incidence for clinically diagnosed anxiety was 1.84%.

In Table 2, the crude ORs for both depressive symptoms (OR 1.85, 95% CI 1.52–2.26) and anxious distress (OR 1.91, 95% CI 1.46–2.49) were significantly higher in the informal caregivers experiencing limitations compared to those that did not give care. These ORs even increased after adjusting for confounding factors for self-reported depressive symptoms (OR 2.00, 95% CI 1.63–2.47) and self-reported anxious distress (OR 2.07, 95% CI 1.57–2.74) in the informal caregivers who experienced limitations compared with those without informal caregiving. When repeating the measurement at the 10-year follow-up, the informal caregivers experiencing limitations still had increased ORs for self-reported depressive symptoms in the crude (OR 1.43, 95% CI 1.07–1.91) and adjusted model (adjusted odds ratio [aOR] 1.44, 95% CI 1.06–1.96) compared to unexposed participants. Furthermore, when adding the baseline self-reported depressive symptoms in the adjusted model, the OR was not significant. There was no significant association between caregiving with limitation and self-reported anxious distress at 10-year follow-up.

**Table 2:** The association between informal caregiving and self-reported depressive symptoms and anxious distress at baseline and 10-year follow-up

	No. of participants	No. of cases	Odds ratios (95% confidence interval)^a^		
			Model 1	Model 2	Model 3
**Baseline (*n*** **= 9346)**					
**Self-reported depressive symptoms**					
No caregiving	8562	1683	1 (ref.)	1 (ref.)	
Caregiving without limitations	312	53	0.83 (0.62–1.12)	0.96 (0.70–1.32)	
Caregiving with limitations	472	146	1.85 (1.52–2.26)	2.00 (1.63–2.47)	
**Self-reported anxious distress**					
No caregiving	8562	714	1 (ref.)	1 (ref.)	
Caregiving without limitations	312	20	0.76 (0.48–1.20)	0.89 (0.55–1.45)	
Caregiving with limitations	472	69	1.91 (1.46–2.49)	2.07 (1.57–2.74)	
**10-year follow-up (*n*** **= 5108)**					
**Self-reported depressive symptoms**					
No caregiving	4626	721	1 (ref.)	1 (ref.)	1 (ref.)
Caregiving without limitations	197	22	0.68 (0.44–1.07)	0.82 (0.52–1.31)	0.86 (0.53–1.40)
Caregiving with limitations	285	60	1.43 (1.07–1.91)	1.44 (1.06–1.96)	1.13 (0.81–1.57)
**Self-reported anxious distress**					
No caregiving	4626	269	1 (ref.)	1 (ref.)	1 (ref.)
Caregiving without limitations	197	10	0.87 (0.45–1.66)	1.04 (0.53–2.03)	1.11 (0.56–2.19)
Caregiving with limitations	285	24	1.49 (0.97–2.31)	1.52 (0.96–2.41)	1.23 (0.76–1.99)

^a^Model 1 was a crude model; model 2 was adjusted for age, sex, social support and socio-economic position; model 3 was additionally adjusted for baseline MDI or rating scale for anxious distress for each outcome respectively

The maximum follow-up time was 16.8 years with an average time to event of 15.5 years for diagnosed depression and 15.6 years for diagnosed anxiety. In Table 3, the HRs of diagnosed depression and anxiety are shown by status of informal caregiving. Informal caregiving with experiencing limitations was associated with clinically diagnosed depression (adjusted HR [aHR] 1.97, 95% CI 1.27–3.05) compared to no caregiving after adjustment of covariates, whereas giving informal care without experiencing limitations (aHR 0.71, 95% CI 0.29–1.74) was not associated with depression. Compared to no informal caregiving, informal caregiving with limitations was associated with an increased HR for anxiety in the adjusted model (aHR 1.86, 95% CI 1.06–3.25), while caregiving without limitations was not associated with anxiety (aHR 0.91, 95% CI 0.33–2.46).

**Table 3:** The association between informal caregiving and clinically diagnosed depression and anxiety by informal caregiving (*n* = 9340^a^)

	No. of participants	No. of cases	Hazard ratio (95% confidence interval)^b^	
			Model 1	Model 2
**Diagnosed depression**				
No caregiving	8558	214	1 (ref.)	1 (ref.)
Caregiving without limitations	312	5	0.64 (0.26–1.56)	0.71 (0.29–1.74)
Caregiving with limitations	470	23	1.98 (1.29–3.04)	1.97 (1.27–3.05)
**Diagnosed anxiety**				
No caregiving	8558	152	1 (ref.)	1 (ref.)
Caregiving without limitations	312	4	0.72 (0.27–1.95)	0.91 (0.33–2.46)
Caregiving with limitations	470	14	1.68 (0.97–2.90)	1.86 (1.06–3.25)

^a^Six participants were excluded because the diagnosis of the outcome occurred before assessment of the exposure

^b^Model 1 was a crude model; model 2 was adjusted for age, sex, social support and socio-economic position

The sensitivity analysis showed increased aHR for diagnosed depression in those caregivers with limitations (aHR 2.07, 95% CI 1.14–3.78) compared to participants not giving care, however not in those giving care but not experiencing limitations (aHR 0.58, 95% 0.14–2.39) (Additional file 1, Table S1). The results for diagnosed anxiety showed nonsignificant HR for informal caregivers without and with limitations (aHR 0.75, 95% 0.18–3.08; aHR 1.70, 95% CI 0.77–3.73), respectively.

## Discussion

We found that caregivers experiencing limitations due to informal caregiving have a 2-fold increased risk of suffering from anxious distress and depressive symptoms compared to non-caregivers, when studied at the same time. Additionally, we found that informal caregivers that experience limitations in their personal life had an increased risk of depression even 10 years later and a higher hazard of clinically diagnosed depression and anxiety. No significant association was found among those informal caregivers without experiencing any limitations. The perceived limitation but not the act of providing care itself seems to be the crucial factor for mental health outcomes.

We found that the prevalence of self-reported depressive symptoms seemed to decline from baseline to follow-up. This might be partly due to the bias of loss to follow-up such that people with depressive symptoms are less likely to participate in the follow-up study. However, we did observe a positive association between caregiving with limitations and clinically diagnosed depression and anxiety. This indicates a long-term effect of caregiving with perceived limitations on caregiver’s mental health. A review by Petrini et al., found a 40% increased risk of depression in family caregivers, which is in line with our findings [6]. This review however, only included people giving care to a family member suffering from dementia and the authors specifically state that the rates of depression in the caregivers increase with a larger cognitive impairment in the family member cared for [6]. While we did not assess cognitive impairment in the persons given care to, this is still an interesting finding and could be taken into account in future studies. In contrast, one study conducted in the Netherlands suggested no association between informal caregiving of a relative and mental ill health.^17^ This discrepancy could be explained by their study having a smaller study population and the exposure definition of informal caregiving which not only included family but also partners and friends.

The suggested underlying reason for mental ill-health in caregivers is stress in particular [23]. Stress due to caregiving comes with high levels of unpredictability and consequences of high stress often affect work, family and relationships [23]. Negative changes in the caregivers lifestyle behaviours include bad dietary habits and little physical exercise which might lead to physical strain [23]. Hence, the possible incompatibility of providing informal care and maintaining a balanced social life as well as upholding working duties increases the risk of mental ill-health, according to the World Health Organisation [3, 18]. Consequently, persons suffering from mental ill-health have been shown to be more susceptible to physical illness [23, 29, 30]. However, a study by Maguire et al. suggested that positive appraisal might reduce the perceived stress and limitations in the caregiver's life, as it shows the appreciation for their caregiving [7].

The strength of this study is the combination of population-based register data and self-administered questionnaire data that were collected longitudinally. By using the different data sources, we were able to include several confounding factors and compare self-reported outcomes with clinically diagnosed outcomes. Even though primary care is not covered by the Swedish medical registers, we were able to detect diagnoses made in primary care, through self-reports of specific questions in the questionnaire. Furthermore, the loss to follow-up was minimised for the longitudinal associations due to the utilisation of register information. No differences between the main analysis and the sensitivity analysis could be detected, which means that loss to follow-up could not have affected our results to any large extent.

This study also faces some limitations. As an analysis on non-respondents showed that participation was associated with having no previous psychiatric inpatient diagnosis [26], non-respondents are more likely to face severe anxiety and depression compared to those that participated. Hence, the external validity of this study is limited, as caregivers with perceived limitations or with mental health problems might not participate. However, this limitation is minimised by utilising the Swedish medical registers in order to check for clinically diagnosed outcomes in those that did not return the follow-up questionnaire. In addition, the informal caregiving was only assessed at baseline and hence, we were unable to adjust for possible changes of caregiving during follow-up periods.

Future research should focus on gender differences in the association between caregiving and perceived as well as diagnosed mental ill-health, as this study did not have enough statistical power to detect differences between genders. Additionally, possible reasons that give rise to the perceived limitations should be further explored.

## Conclusion

This study showed that caregivers experiencing limitations seem to have a higher risk of both short-term and long-term depression and anxiety. The findings imply the importance of exploring the degree to which informal caregiving can be provided without causing any mental problems to caregivers. There is a need for developing additional support strategies to avoid additional health burdens not only on an individual level but also in society at large.

## Supplementary Information

**Additional file 1: Table S1.** The association of informal caregiving with clinically diagnosed depression and anxiety in those who answered the 10-year follow-up questionnaire in wave 3 (*n* = 5106^*^).

## Data Availability

The datasets used and/or analysed during the current study are available from the corresponding author on reasonable request.
