# Supplementary material for: Informal care and the impact on depression and anxiety among Swedish adults: a population-based cohort study
Source: BMC Public Health. 2021 Jun 29;21:1263. doi: 10.1186/s12889-021-11246-1 (PMC8243546; doi:10.1186/s12889-021-11246-1)
Supplement: Supplementary file 1 — Additional file 1: Table S1. The association of informal caregiving with clinically diagnosed depression and anxiety in those who answered the 10-year follow-up questionnaire in wave 3 (n = 5106*). [file 12889_2021_11246_MOESM1_ESM.docx]

**Table S1** The association of informal caregiving with clinically diagnosed depression and anxiety in those who answered the 10-year follow-up questionnaire in wave 3 (*n*=5106^*^)

|  | **No. of participants** | **No. of cases** | **Hazard ratio (95% confidence interval)^#^** | |
| --- | --- | --- | --- | --- |
|  |  |  | **Model 1** | **Model 2** |
| **Diagnosed depression** |  |  |  |  |
| No caregiving | 4625 | 95 | 1 (ref.) | 1 (ref.) |
| Caregiving without limitations | 197 | 2 | 0.49 (0.12-1.98) | 0.58 (0.14-2.39) |
| Caregiving with limitations | 284 | 12 | 2.07 (1.14-3.78) | 2.18 (1.18-4.04) |
| **Diagnosed anxiety** |  |  |  |  |
| No caregiving | 4625 | 77 | 1 (ref.) | 1 (ref.) |
| Caregiving without limitations | 197 | 2 | 0.60 (0.15-2.45) | 0.75 (0.18-3.08) |
| Caregiving with limitations | 284 | 7 | 1.47 (0.68-3.20) | 1.70 (0.77-3.73) |

^*^Two participants were excluded from the data of 10-year follow-up because the diagnosis of the outcome occurred before assessment of the exposure.

^#^Model 1 was a crude model; model 2 was adjusted for age, sex, social support and socio-economic position.
